# Evaluation of NPP-VIIRS Nighttime Light Data for Mapping Global Fossil Fuel Combustion CO_2_ Emissions: A Comparison with DMSP-OLS Nighttime Light Data

**DOI:** 10.1371/journal.pone.0138310

**Published:** 2015-09-21

**Authors:** Jinpei Ou, Xiaoping Liu, Xia Li, Meifang Li, Wenkai Li

**Affiliations:** School of Geography and Planning, and Guangdong Key Laboratory for Urbanization and Geo-simulation, Sun Yat-sen University, Guangzhou, China; University of Maryland at College Park, UNITED STATES

## Abstract

Recently, the stable light products and radiance calibrated products from Defense Meteorological Satellite Program’s (DMSP) Operational Linescan System (OLS) have been useful for mapping global fossil fuel carbon dioxide (CO_2_) emissions at fine spatial resolution. However, few studies on this subject were conducted with the new-generation nighttime light data from the Visible Infrared Imaging Radiometer Suite (VIIRS) sensor on the Suomi National Polar-orbiting Partnership (NPP) Satellite, which has a higher spatial resolution and a wider radiometric detection range than the traditional DMSP-OLS nighttime light data. Therefore, this study performed the first evaluation of the potential of NPP-VIIRS data in estimating the spatial distributions of global CO_2_ emissions (excluding power plant emissions). Through a disaggregating model, three global emission maps were then derived from population counts and three different types of nighttime lights data (NPP-VIIRS, the stable light data and radiance calibrated data of DMSP-OLS) for a comparative analysis. The results compared with the reference data of land cover in Beijing, Shanghai and Guangzhou show that the emission areas of map from NPP-VIIRS data have higher spatial consistency of the artificial surfaces and exhibit a more reasonable distribution of CO_2_ emission than those of other two maps from DMSP-OLS data. Besides, in contrast to two maps from DMSP-OLS data, the emission map from NPP-VIIRS data is closer to the Vulcan inventory and exhibits a better agreement with the actual statistical data of CO_2_ emissions at the level of sub-administrative units of the United States. This study demonstrates that the NPP-VIIRS data can be a powerful tool for studying the spatial distributions of CO_2_ emissions, as well as the socioeconomic indicators at multiple scales.

## Introduction

The increase of global carbon dioxide (CO_2_), which is a major greenhouse gas produced by anthropogenic activities, is the largest positive radiative forcing that contributes to global warming [1]. In order to minimize adverse impacts of climate change, the scientific and policymaking communities have put tremendous efforts into constructing emission inventories. Such inventories can provide quantitative insights into CO_2_ emissions and facilitate the assessment of practical measures for emission reduction [2, 3]. Besides, spatially distributed inventories of carbon emissions can serve as useful input to the global carbon cycle model [4]. Currently, there are several well-known inventories that have provided available estimates of carbon emissions with comprehensive global coverage. For example, the Carbon Dioxide Information Analysis Center (CDIAC) provides national fossil fuel CO_2_ emissions through energy statistics published by the United Nations. The Energy Information Administration (EIA) of the United States Department of Energy (DOE) construct a global inventory of fossil fuel CO_2_ emissions with detail on fuel type (coal, petroleum, and natural gas) derived from a large list of primary energy consumption sources. The International Energy Agency (IEA) generates national fossil fuel CO_2_ with detail on economic sector and derives the information primarily from national energy surveys and emission factors based on Intergovernmental Panel on Climate Change (IPCC) guidelines. The United Nations Framework Convention on Climate Change (UNFCCC) collects national CO_2_ emission estimates with detail on sector, subsector, and fuel. Finally, the Emission Database for Global Atmospheric Research (EDGAR) produced by the JointResearch Centre of the European Commission and the Planbureau voor de Leefomgeving NetherlandsEnvironmental Assessment Agency also provides many emitted species beyond fossil fuel CO_2_ with detail on sector, subsector, and fuel type. In addition to those inventories at the national scale, there has been an increasing emphasis on building global fossil fuel CO_2_ emission data products in gridded form since regularized gridding is particularly useful for use in atmospheric transport models. The CDIAC builds a monthly fossil fuel CO_2_ emission data product on a 1° × 1° grid spanning the time period 1950 to 2010 by downscaling the national emissions with population density. The EDGAR data product provides annual estimates spanning the time period 1990 to 2010 that distributes the national totals into 0.1° × 0.1° grid cells according to a variety of spatial proxies ranging from population density to specific point source location maps. The Open Source Data Inventory of Anthropogenic CO_2_ Emission (ODIAC) generates fossil fuel CO_2_ emissions on a 1 km grid from 1980 to 2007 based on the satellite observations of nighttime lights and a geocoded estimation of power plant CO_2_ emissions. Besides, a recent effort by Rayner et al. [5] constructed global gridded fossil fuel CO_2_ emission quantification through the Fossil Fuel Data Assimilation System (FFDAS) that combined some elements of downscaling, bottom-up information, and data assimilation within a model of fossil fuel CO_2_ emissions to optimally disaggregate national emissions to a 0.25° global grid. From these gridded data products, it can be seen that the development of a global carbon emission inventory requires more accurate and more finely resolved quantification at spatial scale [6].

For constructing high-resolution CO_2_ emission maps on a global scale, satellite-observations of nightlights have been identified as being potentially useful [5, 7]. The nighttime light images, primarily derived from the Defense Meteorological Satellite Program’s (DMSP) Operational Linescan System (OLS), can detect the artificial lights from cities, towns, industrial sites and other human activities at night, thereby providing uniform, spatially explicit, continuous and timely measurements of demographic and economic related activities [8]. Due to this notable advantage, the DMSP-OLS nighttime images have been widely used for assessing economic activity, urban extent and urbanization processes, human population distribution, power consumption, as well as mapping CO_2_ emission distribution [5, 7, 9–12]. For example, Doll et al. [7] revealed that the DMSP-OLS data became an effective tool for global mapping of socioeconomic parameters and greenhouse gas emissions. In another study, Rayner et al. [5] produced a global, annual emission field at 0.25° resolution with various observations, the statistics of national emissions and data on the distribution of nightlights and population.

Although useful, DMSP-OLS nighttime images have a set of well-known limitations related to their coarse spatial resolution (30 arc second, about 1 km), blooming (the “spilling” of light from built-up areas into non-lit areas), saturation in urban areas and intra-sensor calibration problems [13, 14]. These limitations could reduce the correlation between the socioeconomic activity and the nighttime light data [15, 16], resulting in more uncertainties to CO_2_ emission modeling in some areas, especially in the centers of large cities with strong artificial lighting [17, 18]. To deal with the problems, the Earth Observation Group in National Oceanic and Atmospheric Administration’s National Geophysical Data Center (NOAA/NGDC) has provided a global radiance calibrated nightlight product by combining the sparse data acquired at low gain settings with the operational data acquired at high gain settings [19]. Compared with the ordinary DMSP-OLS nighttime light dataset (i.e., the annual cloud-free composited stable lights with a numeric range of 0–63), this radiance calibrated product has fewer saturated pixels and provides a better view of internal characteristics of cities, which is much more suitable for CO_2_ emission modeling. For example, Oda and Maksyutov [20] constructed a global 1 km × 1 km annual fossil fuel CO_2_ emission inventory by combining a worldwide point source database and satellite observations of the global radiance calibrated nightlight distribution. Ghosh et al. [21] also developed a model to allocate the distributed CO_2_ emissions using a combination of DMSP-OLS radiance calibrated nighttime images and population count data. These nightlight-based global CO_2_ emission maps have been improved based on the radiance calibrated data. Unfortunately, only a few of radiance calibrated images are available so far, which is still a challenge for time-series analyses [22].

Recently, a new generation of nighttime light data from the Visible Infrared Imaging Radiometer Suite (VIIRS) carried by the Suomi National Polar-Orbiting Partnership (NPP) satellite was released by the Earth Observation Group in NOAA/NGDC in early 2013 [23, 24]. The NPP-VIIRS nighttime lights were generated using VIIRS day/night band (DNB) data collected on nights with zero moonlight. Compared with DMSP-OLS data, the NPP-VIIRS data feature a higher spatial resolution (15 arc-second, about 500 m). Besides, The NPP-VIIRS data employ onboard calibration, which is not available for the DMSP-OLS data [25–27]. More strikingly, the NPP-VIIRS data do not have the issue of over-saturation existing in the DMSP-OLS data, since VIIRS has a day/night band (DNB) with a spectral range of 500–900 nm that is highly sensitive to very low levels of visible light and can significantly improve the detection ability of anthropogenic lighting [28–29]. In previous studies, some scholars have employed NPP-VIIRS data to estimate the social economy, urban extent and electric power consumption at regional scale, and also demonstrated that NPP-VIIRS nightlight data probably provide higher capacity than that of DMSP-OLS imagery [22]. For example, Li et al. [30] employed NPP-VIIRS data to estimate gross regional products (GRP) in China and demonstrated that the data have a strong capacity in modeling regional economic indicators at the national scale. Similarly, Shi et al. [31] investigated the potential of NPP-VIIRS data in modeling the gross domestic product (GDP) and the electric power consumption (EPC) at multiple scales through a case study of China and revealed that the NPP-VIIRS data can be a powerful tool for modeling socioeconomic indicators. In another study, they also demonstrated that NPP-VIIRS night-time light composite data have better performance in urban built-up area extraction than the DMSP-OLS data [32]. However, to the best of our knowledge, no related work has investigated the potential of NPP-VIIRS nightlight data for estimating CO_2_ emission distribution at a global scale. In addition, there is still a lack of comparison between the CO_2_ emission estimation from NPP-VIIRS data and that from DMSP-OLS data, particularly the radiance calibrated nighttime imagery. To provide a better understanding of the NPP-VIIRS data quality, as well as support further analysis in future research of global warming, a comprehensive evaluation of this new dataset for constructing a global carbon emission inventory is urgently required.

Therefore, this study aims to investigate the potential of NPP-VIIRS data for mapping global fossil fuel combustion CO_2_ emissions with the population distribution dataset. We also conduct a comparative analysis with the stable light data and radiance calibrated data of DMSP-OLS to examine the capability of VIIRS nighttime light. The structure of the paper is organized as follows. A detailed description of data will be presented in Section 2. The model for disaggregating the CO_2_ emissions will be described in Section 3. We will then present the estimation results and discuss the advantages and limits of NPP-VIIRS data in mapping global fossil fuel combustion CO_2_ emissions. Finally, we summarize results and draw conclusions in the last section.

## Data Preparation

### NPP-VIIRS and DMSP-OLS nighttime light imagery

In this study, the only available composite NPP-VIIRS nighttime light data of the year 2012 were obtained from website of NOAA/NGDC (http://ngdc.noaa.gov/eog/viirs/download_viirs_ntl.html). The NPP-VIIRS imagery is a preliminary product, which contains lights from cities, towns, transportation corridors, gas flares, biomass burning and background noise, and in some places has features associated with the reflectance of light from bright surfaces, such as snow covering mountains or bright playa lake beds. Thus, the confounding factors that are irrelevant to socio-economic activities must be removed to improve the accuracy and reliability of CO_2_ emission estimation. So far, a simple and efficient process for removing the confounding factors was adopted based on the hypothesis of Li et al. [30] and Shi et al. [31], which assumes that the lit areas in the NPP-VIIRS data and the DMSP-OLS stable light data of the year 2012 were one in the same. Thus, we generated a mask with all positive value pixels from the DMSP-OLS data in 2012, then overlaid the generated mask with NPP-VIIRS data in 2012 to find the corresponding pixels in the same locations. Those pixels in NPP-VIIRS data were extracted to derive a denoised nighttime light imagery, and the pixels with negative DN values in NPP-VIIRS data were assigned the value of 0. Compared to the preliminary product, the data removing the confounding factors should be more reliable for the estimation [30]. Nevertheless, it is noted that the noise removal method has some deficiencies. For instance, since NPP-VIIRS is at 0.5 km while DMSP-OLS is at about 1km, the valid nightlight pixels of NPP-VIIRS could be dismissed if DMSP-OLS is used as a mask. Also, a small number of the NPP-VIIRS noise that might fall inside the masked area. These deficiencies should be addressed in future research.

To conduct a comparative analysis, the stable light products and radiance calibrated products from DMSP-OLS (hereinafter referred to as ‘SLP-DMSP-OLS’ and ‘RCP-DMSP-OLS’, respectively) were also used in this study. Although the SLP-DMSP-OLS data is not good for estimating CO_2_ emissions due to its major saturation issue at the core of the cities and bright areas, we still bring the SLP-DMSP-OLS in this comparisons for presenting a more comprehensive insight into the differences between three nighttime light imageries in the CO_2_ emission estimations. The SLP-DMSP-OLS data are cloud-free composites which contain lights from cities, towns and other sites with persistent lighting, and have removed ephemeral events (e.g., fires, gas flares, volcanoes and background noise). These products were made using all the available archived DMSP-OLS smooth resolution data for calendar years. Since the NPP-VIIRS data were only available for the year 2012, we used the stable light data in 2012 (F18 satellite, available at http://ngdc.noaa.gov/eog/dmsp/downloadV4composites.html). The RCP-DMSP-OLS data, which were produced by combining images collected at different gain settings (high, medium, and low), were also obtained from the website of NOAA/NGDC (http://ngdc.noaa.gov/eog/dmsp/download_radcal.html). Because the RCP-DMSP-OLS in 2012 are not available so far, we chose the RCP-DMSP-OLS data with an acquisition year close to 2012. This closest available data just characterizes global nighttime lights with less saturated pixels in the year 2010. These nighttime light imageries are all shown in S1 Fig.

### National fossil fuel carbon emissions

In this study, we focused on the disaggregation of national land-based CO_2_ emissions that are attributable to fossil fuel combustion. For this purpose, the national CO_2_ emissions were obtained from the worldwide energy statistics compiled by CDIAC. CDIAC is the primary climate change data and information analysis center of the American Department of Energy that focuses on obtaining, evaluating, and distributing data related to climate change and greenhouse gas emissions, including a continuous archive of national fossil fuel CO_2_ emissions from 1751 to 2012. These CO_2_ emissions data in thousand metric tons were derived from the statistics of fuel (oil, gas, and coal) consumption and cement production, using the methods of Marland and Rotty [33]. Since the radiance calibrated imagery and NPP-VIIRS data were only available for the year 2010 and 2012 respectively, we had to use the national emission data of two years 2010 and 2012 for our analysis.

### Power plant emissions

The national CO_2_ emissions mostly contain the emissions from power plants. Based on the statistical reports from a global power plant database (Carbon Monitoring and Action (CARMA)), power generation accounts for 40% of all CO_2_ emissions in the United States and about one-quarter of global emissions. In particular, there are a number of power plants that generated emissions exceeding 20 Mt CO_2_/year. Thus, the emissions cannot be disaggregated from point sources to global distributed grids [20]. To avoid producing further uncertainty in global emission map, we independently estimated the emissions attributable to power plants by using CARMA (http://carma.org/). CARMA is a database containing information about CO_2_ emissions and locations of over 60,000 power plants worldwide in the years 2004, 2009, and the future (based on planned construction and retirements). The CARMA database does not include the emission data of 2010 and 2012; therefore, in this study, we roughly approximated these data with those of the year 2009, as in the research by Oda and Maksyutov [20]. As shown in S2 Fig, we selected 17,695 power plants (emission > 0) with valid location information and calculated the total power plant emissions for each country. The locations of the top ~1000 emitting power plants and the power plants located in water grid cells were corrected through a combination of visual inspection in Google Earth and additional information provided on individual facility webpages. If the power plant locations could not be confirmed, the emissions were included in the emissions from other sources. In addition, we used the power plant emissions based on the year 2009 to account for the emissions for the year 2010 and 2012. The power plants were assumed to be operational during this period of 2009–2012, and their annual emission levels were simply scaled by the national emission trends obtained from CDIAC. The power plant emissions for the year 2010 and 2012 can be approximated based on the annual emission levels.

By subtracting the power plant emissions from the national total emissions, emissions from other sources in each country were approximated (S1 Table). These sources, which include several sectors such as industry, residence, commerce, and transportation, cause a significant amount of carbon emissions (about three-quarters of the global total). Currently, nightlight and population are the only globally available datasets that can be used to estimate the emissions from these sources. Therefore, in this study the emissions from non-point sources were fittingly adopted for disaggregating into a global distribution.

### LandScan population grid

The LandScan population grid is a progressive series of global population distribution datasets produced by the U.S. Department of Energy (DOE) at Oak Ridge National Laboratory (ORNL) since the late 1990s. Researchers at ORNL used spatial data and imagery analysis technologies, such as multi-variable dasymetric modeling approach, to disaggregate census counts within an administrative boundary [34]. These datasets have a fine resolution of 30 arc-seconds, covering 84° North to 90° South and 180° West to 180° East. The values of the grid cells are integer population counts representing an average or ambient population distribution. The LandScan population grid not only significantly enhances the utility and impact of various applications in estimating ambient population at risk but also widely contributes to urban sprawl detection and greenhouse gas emission evaluation [35]. Therefore, we used the LandScan population grid of 2010 and 2012 for this study. To match the spatial resolution of NPP-VIIRS data, the LandScan population grid of the year 2012 was resampled into a new resolution of 15 arc-second. The value of each grid of new population data was also changed as a quarter of the original one in the corresponding location.

## Method for Mapping CO_2_ Emissions

In this study, we employed a model developed by Ghosh et al. [21] to map CO_2_ emissions (excluding power plant emissions). This model is a top–down process that allocates spatial emission sources from a large geographic area to finer grid cells based on the combination of nighttime lights and population counts. The use of population grid in estimating CO_2_ emissions proved to be advantageous since population data can serve as a proxy for estimating CO_2_ emissions in areas which have no satellite detected lighting [36]. Hence, in this model emissions were distributed in proportion to the brightness of nighttime lights in areas where lighting was detected; In areas without detected lighting, emissions were distributed based on population counts, assuming that each people living in non-illuminated areas emits a half as much CO_2_ as that living in areas with detected lighting [21]. The detailed process of the model is presented as follow:

(1) A mask of the lit areas of the world was created from the nighttime light image. This mask was overlaid on the LandScan population grid and the sum of population from the lit areas of each administrative unit ***i*** was extracted (***SP***_***Li***_). Similarly, a mask of the dark areas of the world was created from the nighttime image and was overlaid on the population grid to extract sum of population of the dark areas of each administrative unit (***SP***_***Di***_).
(2) Assuming that CO_2_ emission per capita for the lit areas of each administrative unit ***i*** is ***x***_***i*,**_ the CO_2_ emission per capita for the dark areas of that administrative unit is ***x***_***i***_ /2. The total CO_2_ emissions from the lit areas (***CO***_***2Li***_) and total CO_2_ emissions from the dark areas (***CO***_***2Di***_) were derived through Eqs 1 and 2, respectively. Since the total CO_2_ emissions of the administrative unit (***TCO***_***2i***_) were the sum of emissions from both the dark and lit areas (Eq 3), the value of the variable ***x***_***i***_ for each administrative unit was obtained from Eq 4.

$${CO}_{2Li}\;=\;{SP}_{Li}\;*\;x_{i}$$ (1)

$${CO}_{2Di}\;=\;{SP}_{Di}\;*\;\left(x_{i}\;/\;2\right)$$ (2)

$${TCO}_{2i}\;=\;{CO}_{2Li}\;+\;{CO}_{2Di}$$ (3)

$$x_{i}\;=\;{TCO}_{2i}\;/\;\left({SP}_{Li}\;+\;{SP}_{Di}\;/\;2\right)$$ (4)

(3) In order to obtain the CO_2_ emissions grid for the lit areas of the administrative unit ***i ***(***CO2Lgi***), each of the lit pixels of the nighttime lights grid (***L***_***pi***_) was multiplied by the CO_2_ emissions per radiance unit which was equal to the ratio of the total CO_2_ emissions from the lit areas (***CO***_***2Li***_) and the sum of light value for that administrative unit (***SL***_***Li***_) (Eq 5). Conversely, the population count in each pixel of the dark areas of the population grid (***P***_***Dpi***_) was multiplied by the CO_2_ emissions per capita, which was derived by the ratio of the total CO_2_ emissions from the dark areas (***CO***_***2Di***_) and the sum of population in the dark areas (***SP***_***Di***_), to distribute the CO_2_ emissions for the dark areas of that administrative unit (***CO2Dgi***) (Eq 6).

$${CO}_{2Lgi}\;=\;L_{pi}\;*\;\left({CO}_{2Li}\;/\;{SL}_{Li}\right)$$ (5)

$${CO}_{2Dgi}\;=\;P_{Dpi}\;*\;\left({CO}_{2Di}\;/\;{SP}_{Di}\right)$$ (6)

(4) These two separate CO_2_ emissions grid from the lit areas and the dark areas of corresponding administrative units ***N*** were added to create the final estimated CO_2_ emissions grid of the world (***CO***_***2g***_) (Eq 7).

$${CO}_{2g}\;=\;\sum_{i=1}^{N}\left({CO}_{2Lgi}\;+\;{CO}_{2Dgi}\right)$$ (7)

It should be noted that the factor with which the nighttime lights pixel should be multiplied to get the CO_2_ emissions per capita from the non-illuminated rural areas is actually a variable since the CO_2_ emissions per capita from the rural areas (corresponding to the darker areas of the nighttime lights image) change from one country to another. Again, for the quarter of the world’s population in darkness, the percentage varies between countries. However, as acknowledged in Ghosh et al. [21], the 0.5 factor was used as a placeholder for demonstrating the CO_2_ production of non-illuminated areas in this model because of the absence of a better known number. Future research will undoubtedly address the uncertainty and produce a map of this parameter that varies from country to country.

## Results and Discussion

### Spatial distribution of CO_2_ emissions

**Fig 1:**
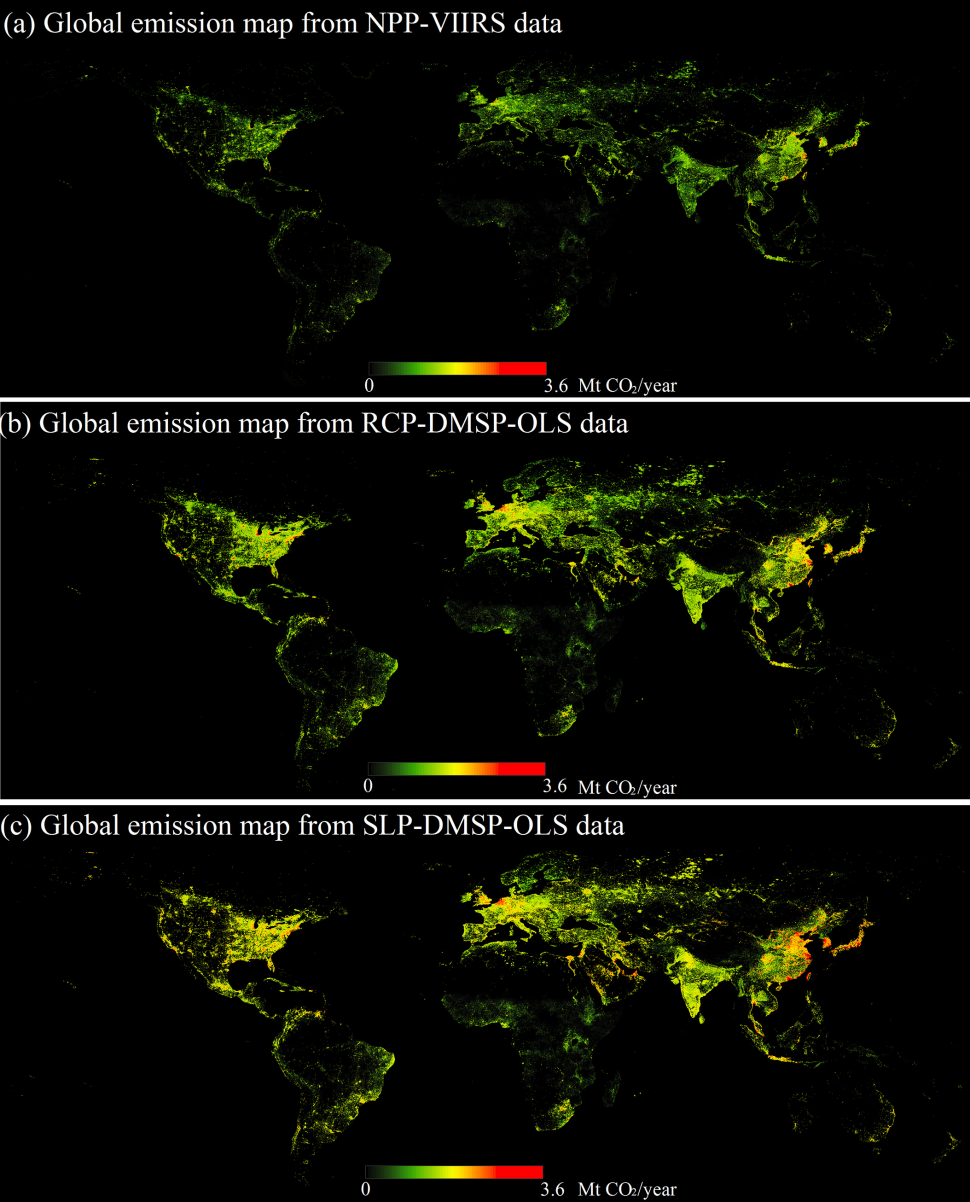
Three global emission maps derived from population counts and three different types of nighttime lights: NPP-VIIRS (a), RCP-DMSP-OLS (b), and SLP-DMSP-OLS (c). The population data is from the U.S. Department of Energy at Oak Ridge National Laboratory (DOE/ORNL), and the three nighttime lights are from the Earth Observation Group in National Oceanic and Atmospheric Administration’s National Geophysical Data Center (NOAA/NGDC).

According to the model described above, the spatial distributions of three gridded global CO_2_ emissions (excluding power plant emissions) were derived from nightlights and population data. The global emission maps from NPP-VIIRS data in 2012 had a spatial resolution of about 0.5 km. While other two maps from RCP-DMSP-OLS data in 2010 and SLP-DMSP-OLS data in 2012, respectively, were both drawn using an about 1 km resolution. As shown in Fig 1, all three global emission maps present massive emissions in Eastern North America, Northern and Western European countries (e.g. UK, Belgium, and the Netherlands), and East Asian countries (e.g. India, China, South Korea, and Japan). In the Southern Hemisphere, in contrast, massive source regions are mainly focused along the coast, which are not as prevalent as in the Northern Hemisphere. In addition, the differences of emission characteristics can be distinguished among the three global emission maps. From Fig 1, it is easy to find that the most widely distributed CO_2_ emissions are visible in the global emission map from SLP-DMSP-OLS data, whereas the spatial distributions of global emission map from NPP-VIIRS data depict less apparent compared to other two maps from DMSP-OLS data. This could be resulted from the blooming and oversaturation limitations of DMSP-OLS nighttime light imagery.

**Fig 2:**
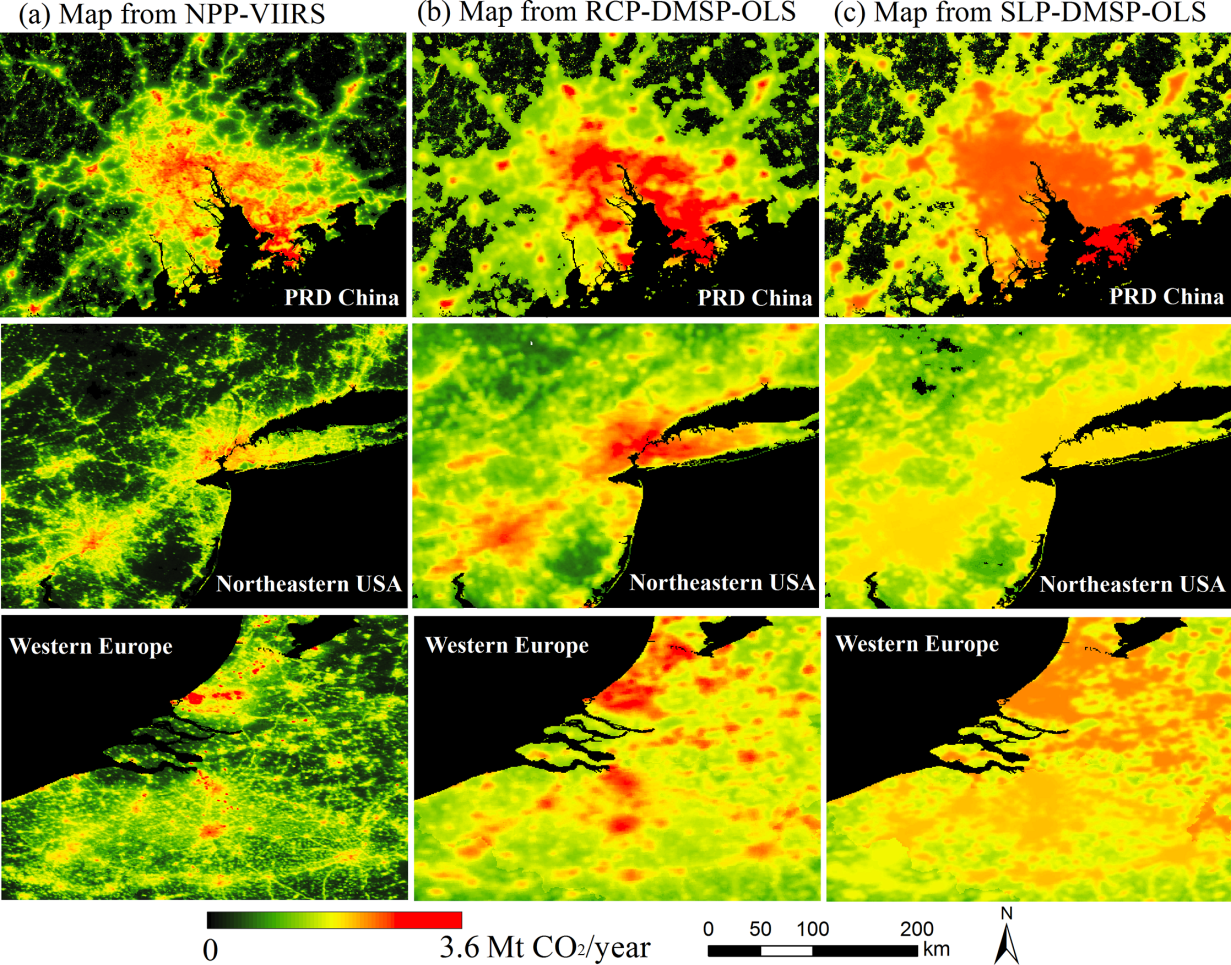
Regional spatial distributions of CO_2_ emissions in the PRD China (a), Northeastern USA (b), and Western Europe (c).

At the regional scale, a fine depiction of spatial emission features of three emission maps is also presented in the enlarged views of the Pearl River Delta (PRD) in China, Northeastern USA and Western Europe (Fig 2). In the map from NPP-VIIRS data, the spatial variability of CO_2_ emission levels could be clearly seen in city cores, particularly at the region of PRD China. In contrast to other two emission maps, the map from SLP-DMSP-OLS data presents wider emission distributions at three regions but much lower emission intensities in city cores, which could be the contribution of blooming and saturation limitations. For the map from RCP-DMSP-OLS data, the depiction of emission sources does not present as clearly as that in the map from NPP-VIIRS data. For example, emission sources along the interstate highway networks are visible spatial characteristics in the map from NPP-VIIRS data, but difficult to be identified in the map from RCP-DMSP-OLS data. The visual observations in Fig 2 indicate that the global emission map from NPP-VIIRS data exhibits a more reasonable emission distribution than other two maps from DMSP-OLS data.

### Comparison with the land cover dataset at city-level scale

In general, the fossil fuel CO_2_ emissions primarily come from urban areas due to human activities. While other areas, such as cultivated land, forest, grassland, and water body, are not the important sources of fossil fuel CO_2_ emissions. By analyzing the spatial consistency between the land covers and emission distributions, we can assess the performance of these global emission maps. For example, if most emissions are found to be distributed in the water bodies through an overlay analysis, we can expect that this emission map produces a poor performance in the spatial distributions of emission estimations, because it is not reasonable to assume that a large amount of energy consumptions can occur in the water bodies. Therefore, we used the land covers as the reference data to examine the differences of spatial distri1butions among these three global emission maps. The land cover data was acquired from a product of GlobeLand30, which was released by National Geomatics Center of China in 2014 (http://www.globallandcover.com). This product, as the world’s first global land cover dataset with a 30m resolution in the years 2000 and 2010, is organized by ten major land cover classes and can provide essential high resolution land cover and change information for climate change studies, environment monitoring, and many other societal benefit areas. Considering that the temporal difference between land cover dataset of 2010 and nighttime light data of 2012 is slight, we believe that a comparison between them can be used to analyze the spatial distributions of three global emission maps. In addition, due to limited space of the letter, we only selected three typical cities (Beijing, Shanghai, and Guangzhou) for this comparison. The land covers of these three cities were categorized into six types, namely cultivated land, forest, grassland, water body, artificial surfaces and others.

**Fig 3:**
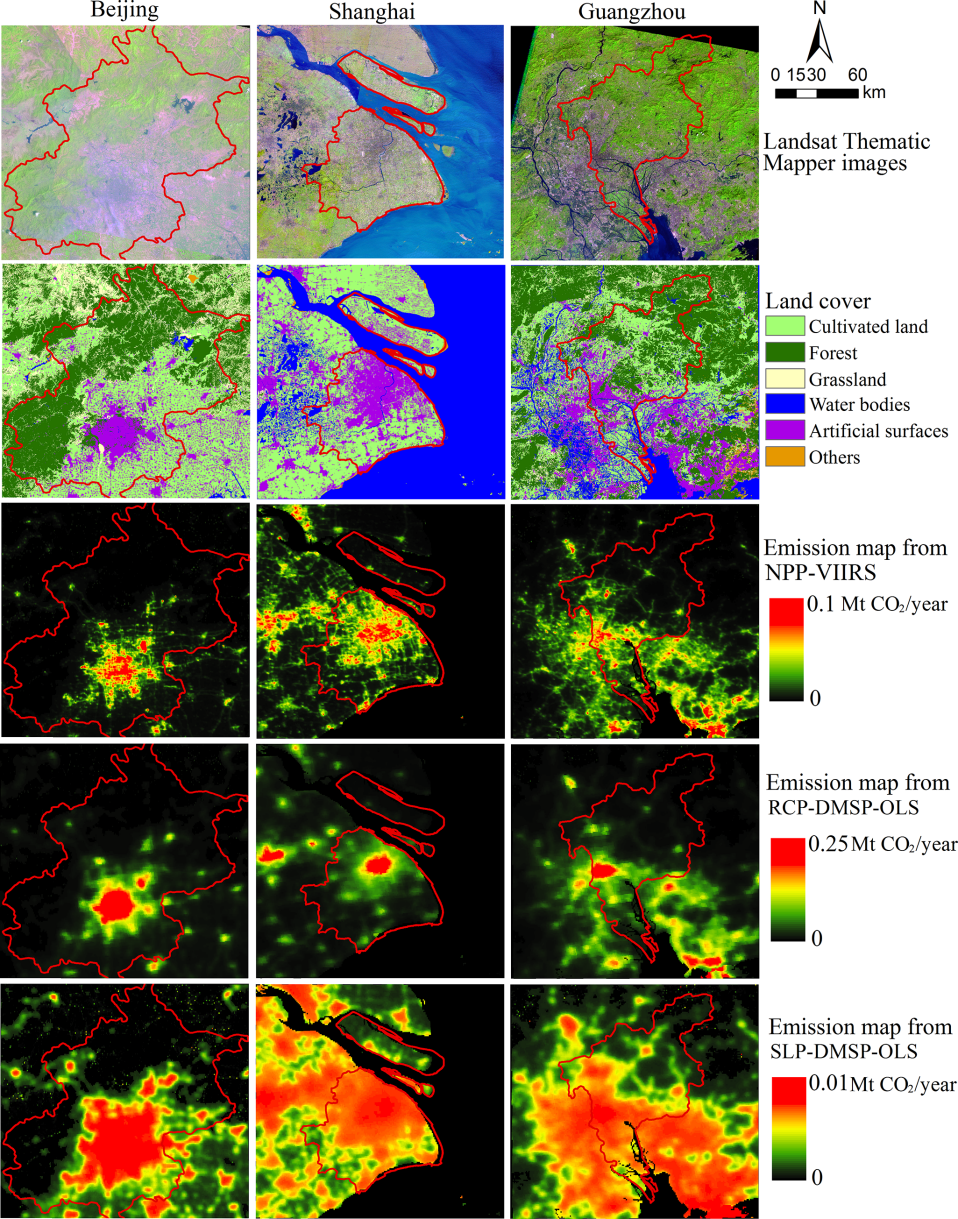
The original Landsat Thematic Mapper images, land cover dataset, and three global emission maps of three cities (Beijing, Shanghai, and Guangzhou). The Landsat Thematic Mapper images of these cities are from the United States Geological Survey (USGS), and the land cover dataset is from the National Geomatics Center of China (NGCC).

The land cover dataset, the original Landsat Thematic Mapper images and three global emission maps of three cities were illustrated in Fig 3. In the emission map from NPP-VIIRS data, the emission areas of the three cities have higher spatial consistency of the artificial surfaces than those of other two maps from DMSP-OLS data, which implies that the map from NPP-VIIRS data delineates spatial patterns of CO_2_ emissions more exactly. Similar to emission map from NPP-VIIRS data, the emission distributions of map from RCP-DMSP-OLS data mostly focus on the artificial surfaces of the three cities. However, the spatial variability in CO_2_ emission levels could be seen even in city cores, which is hard to reflect human activities and to support a more accurate CO2 emission estimation. For the map from SLP-DMSP-OLS data, the emission areas of the three cities spread out the artificial surfaces considerably. Even the regions of water bodies and urban forests in the three cities are also distributed with the emissions in the map from SLP-DMSP-OLS data. This overestimation in the emission distributions could be owing to both the blooming effect and the coarse spatial resolution in SLP-DMSP-OLS data.

**Fig 4:**
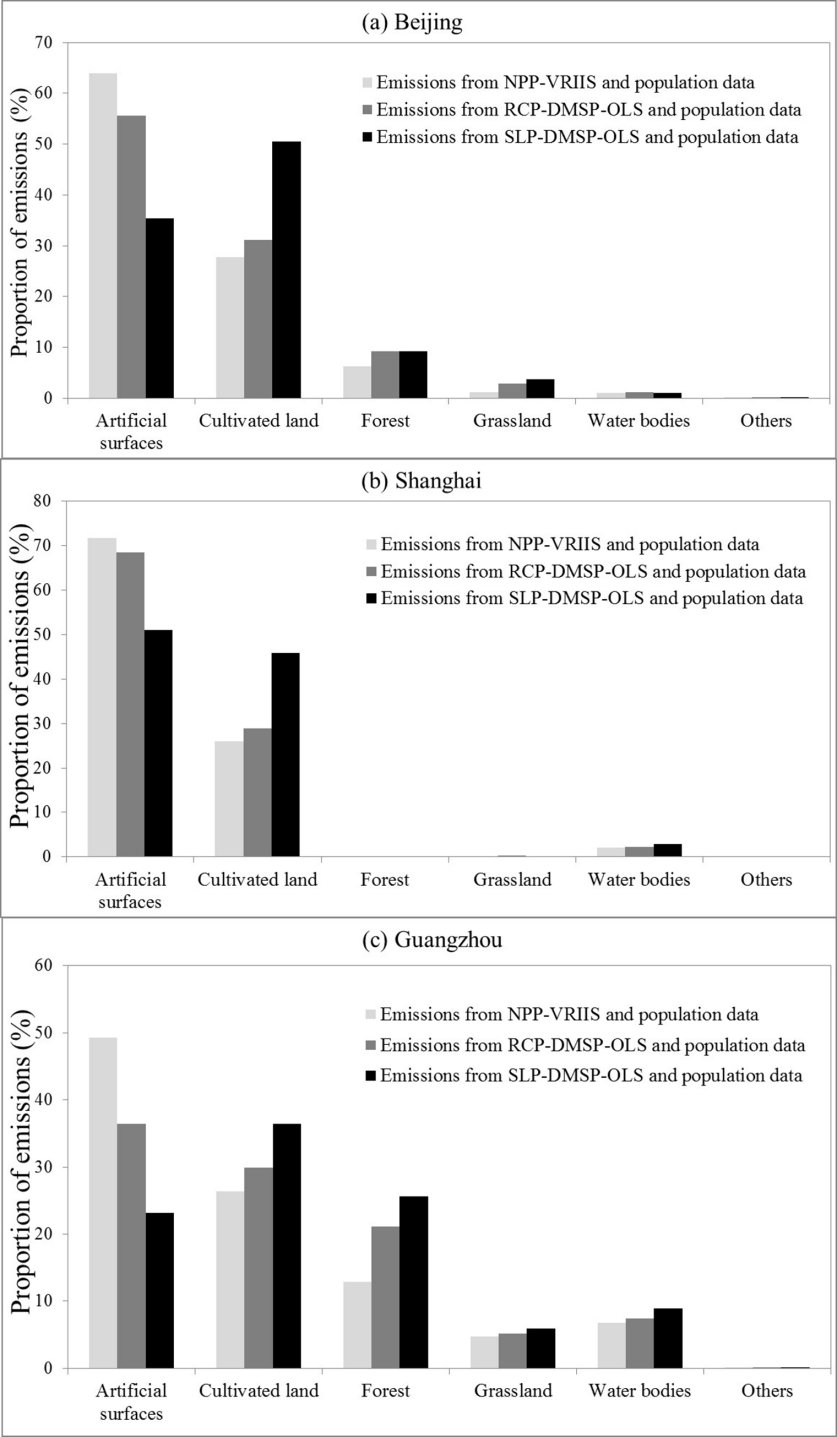
The proportions of CO_2_ emissions in each land cover of the three cities: Beijing (a), Shanghai (b), and Guangzhou (c).

In addition, the proportions of CO2 emissions distributed in each land cover are also calculated to evaluate the difference in the spatial patterns of three emission maps quantitatively. As seen in Fig 4, a majority of CO2 emissions from NPP-VRIIS data are distributed in the artificial surfaces of the three cities. Compared to the emissions from RCP-DMSP-OLS and SLP-DMSP-OLS data, the proportions of emissions from NPP-VRIIS data are much higher in the artificial surfaces, but lower in other land covers. This indicates that the spatial patterns of emissions from NPP-VRIIS data are more consistent with the land cover data. Conversely, in the artificial surfaces, the proportions of emissions from SLP-DMSP-OLS data are the lowest one among the three emission maps. Moreover, in Beijing and Guangzhou, most of the emissions from SLP-DMSP-OLS data are distributed in the cultivated land. These proportions of CO2 emissions also demonstrate that there exists a serious blooming effect in the emission map from SLP-DMSP-OLS data.

**Fig 5:**
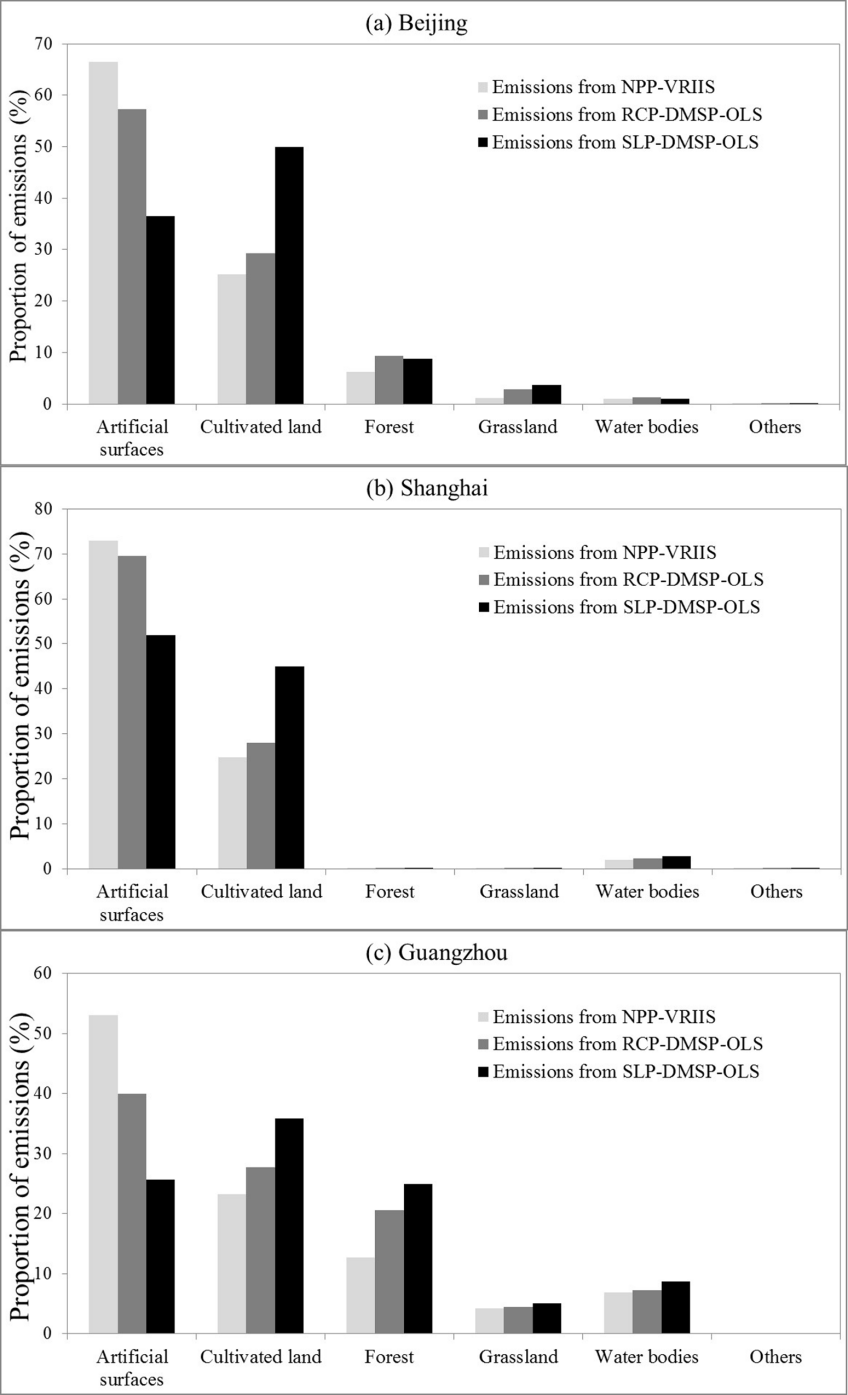
The proportions of nightlight-based emissions distributed over each land cover of the three cities: Beijing (a), Shanghai (b), and Guangzhou (c).

To investigate the utility of NPP-VIIRS data for mapping CO_2_ emissions, we also subtracted the population-based emissions of these three cities and evaluated the proportions of CO2 emissions only distributed by nighttime lights in this comparison. As shown in Fig 5, the proportions of the nightlight-based emissions distributed over each land cover are similar with the previous results (Fig 4). A majority of CO_2_ emissions only distributed by NPP-VRIIS data are still focus on the artificial surfaces of the three cities compared to the emissions from RCP-DMSP-OLS and SLP-DMSP-OLS data. Also, most of the emissions from SLP-DMSP-OLS data are distributed in the cultivated land of these three cities. It can be seen that the global emission map only distributed by NPP-VIIRS data also shows a higher accuracy in the spatial distribution of CO_2_ emission than the other map from only RCP-DMSP-OLS or SLP-DMSP-OLS data.

Therefore, from the comparisons with the land cover dataset, the NPP-VIIRS nighttime light data have a better performance in mapping the global emissions than RCP-DMSP-OLS and SLP-DMSP-OLS data. The better estimated results mainly benefit from the higher spatial resolution and wider radiometric detection range of NPP-VIIRS nighttime light data.

### Comparison to Vulcan inventory

To assess the ability of NPP-VIIRS data in constructing global CO2 emission inventories, an accuracy assessment of these three emission maps is urgently needed. However, it is noted that such assessment is challenging to perform primarily because no global actual measurement data verifying the true distribution of global CO2 emission are available [20]. Although there have been several satellites such as the Greenhouse gases Observing Satellite (GOSAT) and Orbiting Carbon Observatory -2 (OCO-2) that can measure the CO_2_ concentration in the atmosphere at coarse spatial resolution, the CO_2_ signals from satellites are diffused and spatially offset from the sources due to the coarse spatial resolution, atmospheric transport, mixing, and retention of CO_2_ in the atmosphere [21]. It is difficult to discern increases or decreases in CO_2_ emissions from specific cities or towns and to validate the spatial characteristics of high-resolution emission maps with these data from satellites. Fortunately, Gurney et al. [37] have produced the Vulcan fossil fuel emissions data product from a bottom-up perspective and offered a useful point of comparison at the regional scale. The Vulcan product provides fossil fuel CO_2_ emissions for the U.S. on a 0.1° grid with a temporal resolution of 1 h and includes process-level detail such as combustion technology, fuel type, and vehicle class. So far Vulcan is most accepted bottom up dataset which is not using proxies to distribute emissions. All the rest of the inventories such as ODIAC, FFDAS and PKU have compared their gridded dataset with Vulcan. Thus, in this study we used the Vulcan emissions data product as a standard to evaluate the three global emission maps (S3 Fig). Although the Vulcan product is only available in the year of 2002, it can still validate these three global emission maps to a certain agreeable degree since there is a small change in the U.S. CO_2_ emissions from 2002 to 2012 (EIA, http://www.eia.gov/environment/emissions/carbon/).

Following Rayner et al. [5], we firstly aggregated all global emission maps into a 0.1° × 0.1° grid scale to match the spatial resolution of Vulcan product. Furthermore, we choose two metrics which reflect likely uses for this comparison: (1) summed absolute difference (SAD, in units of Mt CO_2_/year), which is the sum of the absolute difference of the field over the domain and (2) spatial correlation (SC), which quantifies the magnitude-independent correspondence of the spatial patterns. Finally, considering the influence likely caused by population distribution, we also show the comparison for CO_2_ emission maps distributed by nighttime lights alone.

The results of these comparisons are shown in Tables 1 and 2. From Table 1, we can find that the value of SAD for the estimated emissions from NPP-VIIRS and population data is the lowest in these emission maps. Also, the value of SC for this emission map is substantially higher than other estimates. This suggests that the emission map based on NPP-VIIRS and population is closer to the Vulcan inventory than other emission maps. In contrast, the emission map based on SLP-DMSP-OLS and population data produces the worst performance in this comparison, with the SAD value of 3554.12 Mt CO_2_/year and the SC value of 0.76. This worst result is probably caused by the serious blooming effect and saturation issue of SLP-DMSP-OLS nighttime light data. For the emission map based on RCP-DMSP-OLS and population, it gets a secondary value of SAD and SC, displaying the performance intermediate between the two former maps. Similarly, the comparisons in which only nightlights are used as the spatial proxy reached the same conclusions as the CO_2_ emission maps distributed by nighttime lights and population data. From Table 2, we also see that the estimated emissions only distributed by NPP-VIIRS exhibits the lowest summed absolute differences (3114.24 Mt CO_2_/year) and the highest correlation value (0.85), revealing that it has the best calibration to the Vulcan data product among these nightlight-based estimates. Thus, by the evaluation of the global emission maps versus the Vulcan data product in the USA, the NPP-VIIRS nighttime light data is superior to RCP-DMSP-OLS and SLP-DMSP-OLS data in mapping the global emissions.

**Table 1:** Comparison of the global emission maps based on nightlight and population data to the Vulcan inventory for U.S. domain at the 0.1° resolution.

Metric	NPP-VIIRS and population	RCP-DMSP-OLS and population	SLP-DMSP-OLS and population
SAD	3114.24	3347.1	3554.12
SC	0.85	0.81	0.76

**Table 2:** Comparison of the global emission maps only distributed by nightlights to the Vulcan inventory for U.S. domain at the 0.1° resolution

Metric	NPP-VIIRS	RCP-DMSP-OLS	SLP-DMSP-OLS
SAD	3368.35	3673.54	3751.74
SC	0.83	0.8	0.753

### Accuracy assessment with the statistical data of CO2 emissions

In addition, we performed a quantitative comparison of three global emission maps with the actual statistical data of CO_2_ emissions at the level of sub-administrative units. The statistical data of CO_2_ emissions of sub-administrative regions, which can be gathered and measured by research institutions, are generally regarded acceptable and accurate. Thus the statistical data was used as a standard to evaluate the performances of NPP-VIIRS, RCP-DMSP-OLS and SLP-DMSP-OLS data. Considering the limitation and reliability of data sources, we only adopted the statistical data of sub-administrative regions from the United States for this comparison (S2 Table). The data of CO_2_ emissions of each state in the United States was based on energy consumption data from EIA (http://www.eia.gov/environment/emissions/state/). By subtracting the emissions of power plants, the actual total emissions of each state in 2010 and 2012 were approximated. After that, we aggregated the emissions in each pixel of three emission maps to the level of sub-administrative units and compared them to the statistics of CO_2_ emissions using the regression analysis. The R^2^ of regression analysis and mean relative error (MRE) were used to evaluate the agreement between the actual statistics and aggregated estimated emissions from nighttime lights.

**Fig 6:**
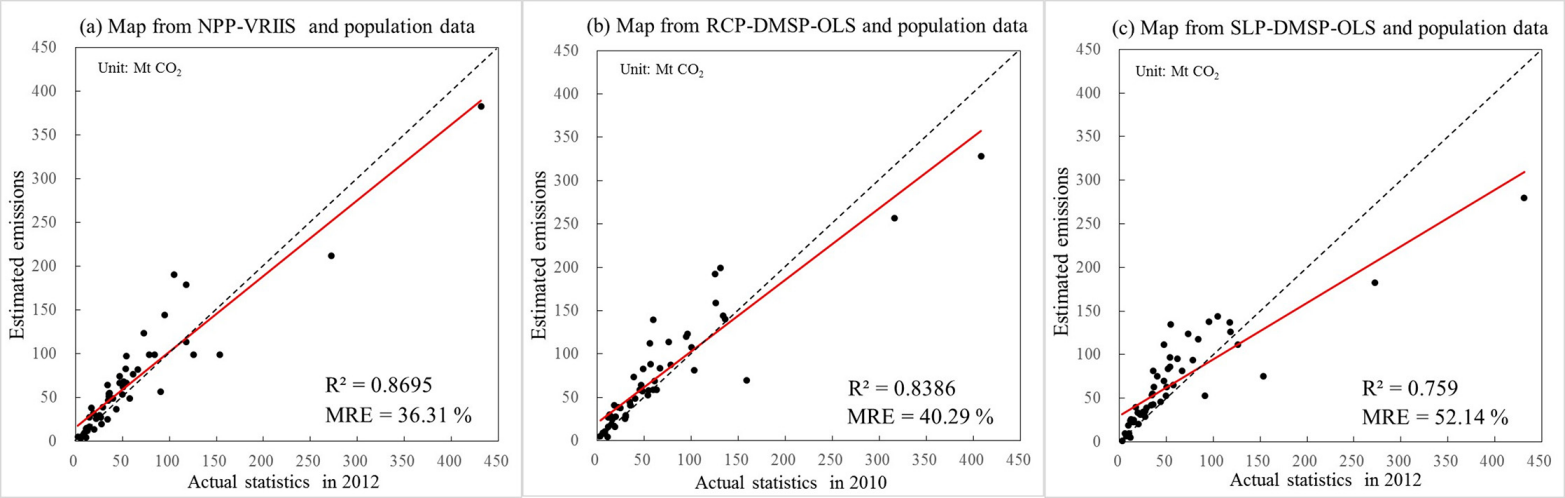
Comparison between the actual statistical data of CO_2_ emissions and the estimated emissions of different emission maps at the state unit of the United States.

The regression results from Fig 6 suggest that the total estimated emissions from nighttime lights and population data have a positive linear relationship with actual statistics in each state of the United States. The R^2^ values of estimated emissions from RCP-DMSP-OLS and SLP-DMSP-OLS data were 0.8386 and 0.759, respectively, both of which are less than that of the estimated emissions from NPP-VIIRS data (R^2^ = 0.8695). In addition to the difference in R^2^ values, the MRE values are also different among all the estimated results. The MRE of estimated emissions from NPP-VIIRS data is 36.31%, whereas those of estimated emissions from RCP-DMSP-OLS and SLP-DMSP-OLS data are 40.29% and 52.14%, respectively. This result indicates that the estimated emissions from NPP-VIIRS data are better fit with the actual statistical data and are more accurate than the other two estimated emissions from DMSP-OLS data at the state unit of the United States.

**Fig 7:**
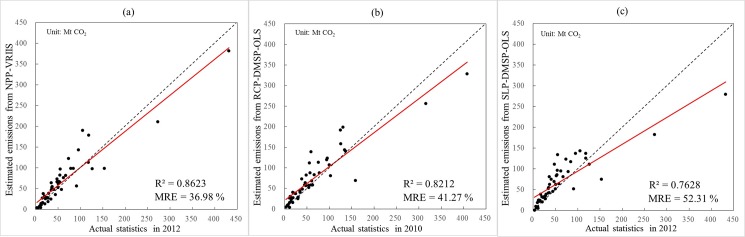
Comparison of emission results only distributed by three different types of nighttime lights at the state unit of the United States: NPP-VIIRS (a), RCP-DMSP-OLS (b), and SLP-DMSP-OLS (c).

Given that the population-based emissions may affect the results, we also attempted to create the emission maps only distributed by nighttime lights and compared them with the actual statistical data of CO_2_ emissions at the state unit of the United States. As shown in Fig 7, there is a small change in the R^2^ and MRE values compared to the previous results. However, the correlation between the actual statistics and aggregated estimated emissions only distributed by NPP-VIIRS data is still stronger than that from RCP-DMSP-OLS or SLP-DMSP-OLS data. Moreover, the estimated emissions from NPP-VIIRS data show the strongest response to the actual statistics with the minimum value of MRE (36.98%). The estimated emissions only distributed by NPP-VIIRS data also have better agreement with the actual statistics at the state level of United States compared to RCP-DMSP-OLS and SLP-DMSP-OLS data. Therefore, based on the comparative analysis of R^2^ values and MRE, we can confirmed that the NPP-VIIRS data is more reliable in estimating spatial distribution of global CO2 emission than the DMSP-OLS data.

### Uncertainties behind the results

Because of higher spatial resolution and increased low-light sensing capability compared with DMSP sensor, the NPP-VIIRS data was proven to derive a better spatial distribution of global CO2 emission inventory. However, we also acknowledge that possible uncertainties exist in our gridded emission inventory. These uncertainties are largely due to problems with data quality or availability. First, the NPP-VIIRS nighttime light data released by NOAA/NGDC are raw data in which fires, gas flares, volcanoes, and other background noise have not been removed. Although the correction method has been applied to the data in this study, the negative effects of some confounding factors still obstruct a better estimation of CO2 emission from NPP-VIIRS data. For instance, since NPP-VIIRS is at 0.5 km while DMSP-OLS is at about 1km, the valid nightlight pixels of NPP-VIIRS could be dismissed if DMSP-OLS is used as a mask. Also, a small number of the NPP-VIIRS noise that might fall inside the masked area. Thus, the background noises need to be removed to improve the quality of NPP-VIIRS data using some advanced techniques. Second, the use of a point-source database (CARMA) is an appealing feature of the present study. However, CARMA database obviously has a number of limitations—the database does not cover all existing power plants worldwide and the geographical coordinates of power plants sometimes indicate false locations because of errors in deriving coordinate information [38, 39]. In addition, we roughly approximated the CARMA emissions for the year 2010 and 2012 with those of the year 2009, as in the research by Oda et al. [20]. Therefore, uncertainties would arise because of the approximation of emissions. Besides, the Landscan is a product based on statistical data and satellite data analysis. The errors also go to the analysis in this study. Finally, these comparisons which focused on only America are deficient in the global emission estimation since the country cannot be representative of the whole world. This would result in the lack of reliability in validating the global emission inventory due to the limitation and absence of the sub-administrative statistics of CO_2_ emissions in other countries. Thus, further comparisons that could show the differences in other countries should be carried out in emission estimation as much as possible.

## Conclusions

In this study, we investigated the ability of NPP-VIIRS data to estimate the spatial distribution of gridded global CO_2_ emissions (excluding power plant emissions) for the first time. Through a top–down model that allocates spatial emission sources from a large geographic area to finer grid cells, three global emission maps were derived from population counts and three different types of nighttime lights (NPP-VIIRS, RCP-DMSP-OLS and SLP-DMSP-OLS), respectively. The comparison with reference data of land cover shows that the global emission map from NPP-VIIRS data have a larger quantity and cleaner spatial variation of CO_2_ emission in the artificial surfaces than the maps from RCP-DMSP-OLS and SLP-DMSP-OLS data, although there is a two-year gap between the NPP-VIIRS data and land cover dataset. In addition, from the evaluation of the global emission maps versus the Vulcan data product and the accuracy assessment with the statistical data of CO_2_ emissions at the sub-administrative units of the United States, the comparison results also demonstrate that NPP-VIIRS nighttime light data is more powerful and reliable than RCP-DMSP-OLS and SLP-DMSP-OLS data in estimating the spatial distributions of CO_2_ emissions.

In conclusion, our analysis revealed that the NPP-VIIRS data can be used as important data source for studying the spatial distributions of CO_2_ emissions. Since there are some challenging problems such as the background noise in NPP-VIIRS nightlight data, further investigations are required in order to improve the quality of the imagery. Besides, future study can be taken on multi-temporal analysis of the imagery in wider fields as more and more NPP-VIIRS nighttime light data are produced.

## Supporting Information

S1 Fig Three different nighttime light imagery: NPP-VRIIS (a), the stable light products (b) and radiance calibrated products (c) from DMSP-OLS. Source: Earth Observation Group in National Oceanic and Atmospheric Administration’s National Geophysical Data Center (NOAA/NGDC).
(TIF)

S2 Fig Global spatial distributions of power plant emissions. Source: Carbon Monitoring and Action (CARMA).
(TIF)

S3 Fig Comparison of CO2 emission estimates for U.S. domain at 0.1 degree grid scale. (TIF)

S1 Table National emissions excluding power plant emissions in 2010 and 2012 (Mt CO_2_). (DOCX)

S2 Table State energy-related carbon dioxide emissions in 2010 and 2012 (Mt CO_2_). Source: U.S. Energy Information Administration (EIA).
(DOCX)
