# Supplementary figures and images for: Evaluation of NPP-VIIRS Nighttime Light Data for Mapping Global Fossil Fuel Combustion CO2 Emissions: A Comparison with DMSP-OLS Nighttime Light Data
Source: PLoS One. 2015 Sep 21;10(9):e0138310. doi: 10.1371/journal.pone.0138310 (PMC4577086; doi:10.1371/journal.pone.0138310)

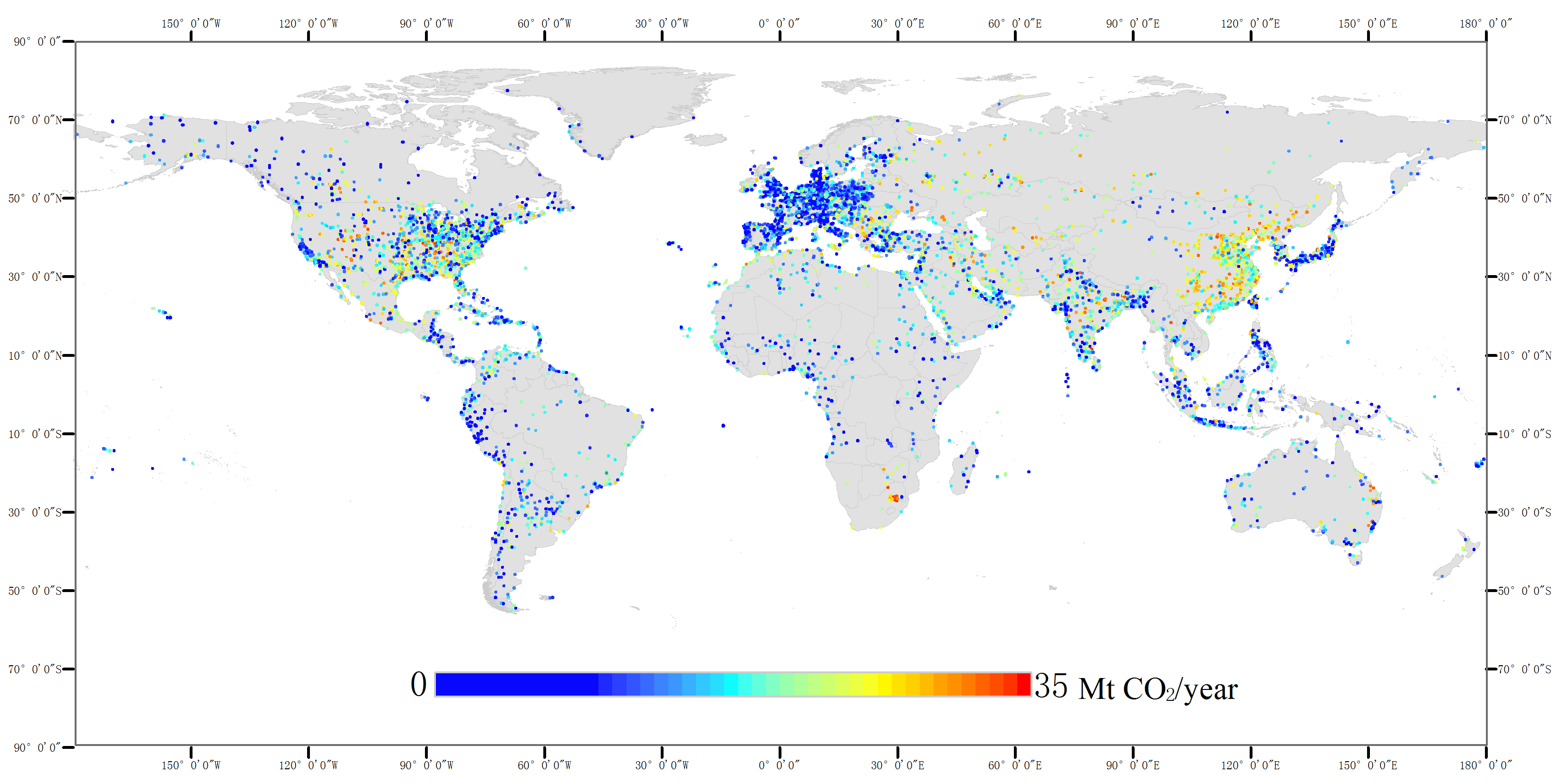

Supplement: S2 Fig — Source: Carbon Monitoring and Action (CARMA). (TIF) [file pone.0138310.s002.tif]

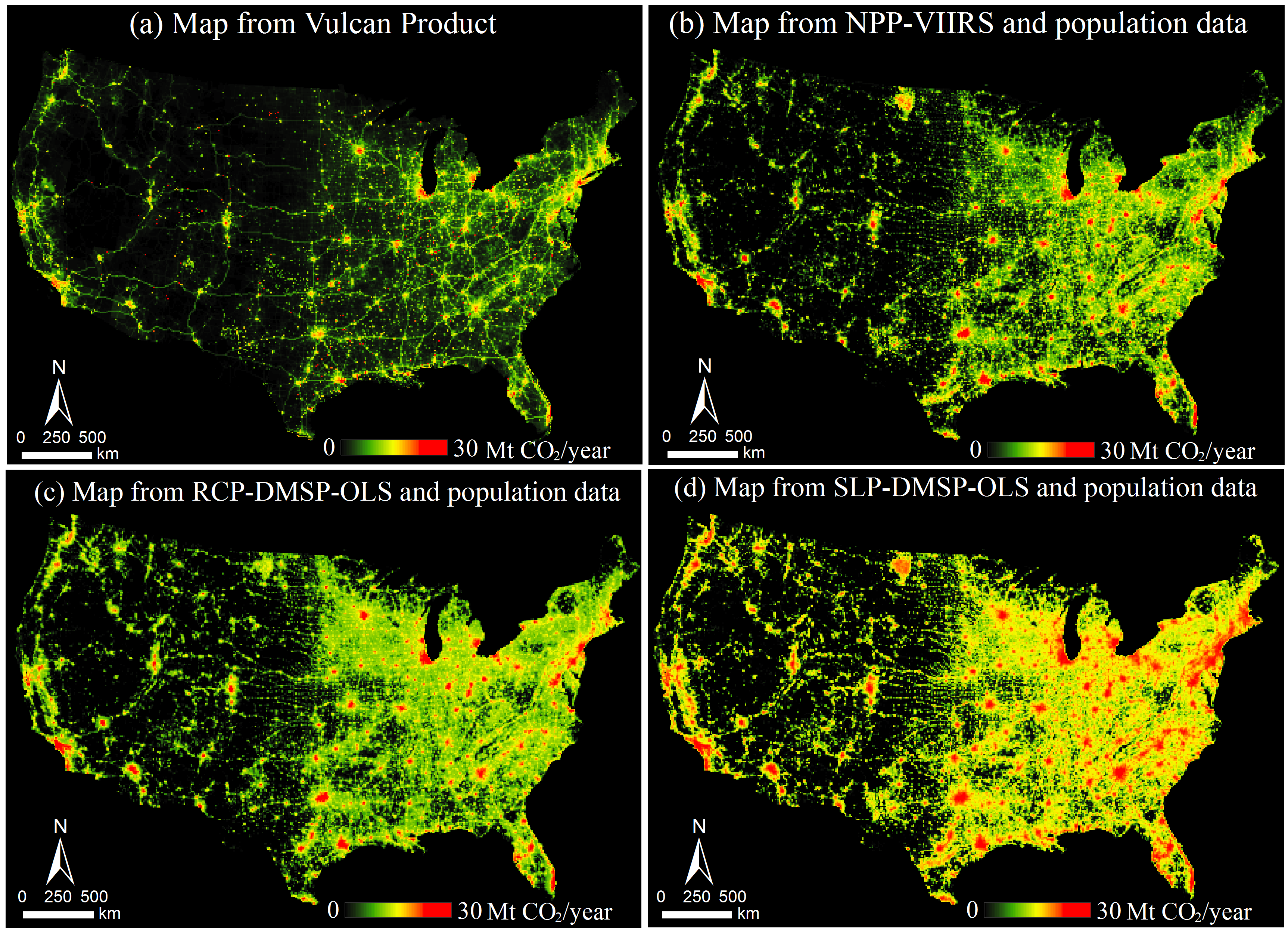

Supplement: S3 Fig — (TIF) [file pone.0138310.s003.tif]
