# Supplementary material for: Evaluation of NPP-VIIRS Nighttime Light Data for Mapping Global Fossil Fuel Combustion CO2 Emissions: A Comparison with DMSP-OLS Nighttime Light Data
Source: PLoS One. 2015 Sep 21;10(9):e0138310. doi: 10.1371/journal.pone.0138310 (PMC4577086; doi:10.1371/journal.pone.0138310)
Supplement: S1 Table — (DOCX) [file pone.0138310.s004.docx]

S1 Table. National emissions excluding power plant emissions in 2010 and 2012 (Mt CO_2_).

| **Country** | **2010** | **2012** | **Country** | **2010** | **2012** |
| --- | --- | --- | --- | --- | --- |
| Austria | 61.30 | 53.96 | US | 3814.71 | 3470.48 |
| Azerbaijan | 17.06 | 22.30 | Canada | 509.64 | 518.45 |
| Belarus | 52.40 | 55.48 | Mexico | 344.50 | 381.79 |
| Belgium | 134.76 | 120.60 | Argentina | 151.82 | 167.12 |
| Bulgaria | 19.72 | 20.61 | Brazil | 449.47 | 477.75 |
| Czech Republic | 69.35 | 60.84 | Chile | 52.91 | 72.05 |
| Denmark | 36.18 | 26.03 | Colombia | 62.99 | 67.12 |
| Finland | 36.26 | 24.87 | Ecuador | 30.24 | 32.51 |
| France | 360.18 | 339.41 | Peru | 34.59 | 42.27 |
| Germany | 535.69 | 517.01 | Trinidad & Tobago | 49.75 | 45.80 |
| Greece | 44.43 | 37.82 | Venezuela | 159.39 | 165.69 |
| Hungary | 39.60 | 34.91 | Other S. & Cent. America | 142.53 | 150.91 |
| Republic of Ireland | 30.32 | 25.24 | Iran | 482.16 | 510.46 |
| Italy | 310.33 | 275.03 | Israel | 56.80 | 63.52 |
| Kazakhstan | 99.87 | 126.85 | Kuwait | 52.67 | 56.04 |
| Lithuania | 15.09 | 15.15 | Qatar | 56.71 | 73.22 |
| Netherlands | 223.15 | 204.79 | Saudi Arabia | 458.54 | 509.37 |
| Norway | 42.26 | 42.87 | United Arab Emirates | 183.85 | 196.31 |
| Poland | 189.26 | 177.21 | Other Middle East | 172.97 | 293.69 |
| Portugal | 36.97 | 36.05 | Australia | 177.79 | 177.15 |
| Romania | 51.48 | 53.85 | Bangladesh | 38.16 | 46.94 |
| Russian Federation | 1176.62 | 1235.95 | China | 5475.26 | 6738.12 |
| Slovakia | 31.66 | 30.81 | China Hong Kong SAR | 58.76 | 61.53 |
| Spain | 238.75 | 251.88 | India | 994.54 | 1169.78 |
| Sweden | 54.31 | 46.17 | Indonesia | 348.32 | 395.13 |
| Switzerland | 42.19 | 41.47 | Japan | 1002.71 | 1095.71 |
| Turkey | 211.41 | 227.64 | Malaysia | 166.39 | 166.51 |
| Turkmenistan | 50.42 | 52.84 | New Zealand | 30.03 | 31.01 |
| Ukraine | 237.89 | 259.56 | Pakistan | 127.06 | 128.31 |
| United Kingdom | 386.65 | 358.94 | Philippines | 51.40 | 57.62 |
| Uzbekistan | 81.27 | 87.51 | Singapore | 189.44 | 199.26 |
| Other Europe & Eurasia | 194.98 | 200.42 | South Korea | 499.71 | 549.79 |
| Algeria | 83.03 | 98.37 | Taiwan | 219.98 | 215.19 |
| Egypt | 159.59 | 172.48 | Thailand | 241.80 | 273.78 |
| South Africa | 220.42 | 221.60 | Vietnam | 108.33 | 117.14 |
| Other Africa | 338.74 | 350.41 | Other Asia Pacific | 111.70 | 121.86 |
