# Supplementary material for: Evaluation of NPP-VIIRS Nighttime Light Data for Mapping Global Fossil Fuel Combustion CO2 Emissions: A Comparison with DMSP-OLS Nighttime Light Data
Source: PLoS One. 2015 Sep 21;10(9):e0138310. doi: 10.1371/journal.pone.0138310 (PMC4577086; doi:10.1371/journal.pone.0138310)
Supplement: S2 Table — Source: U.S. Energy Information Administration (EIA). (DOCX) [file pone.0138310.s005.docx]

S2 Table. State energy-related carbon dioxide emissions in 2010 and 2012 (Mt CO_2_). Source: U.S. Energy Information Administration (EIA).

| **State** | **2010** | **2012** | **State** | **2010** | **2012** |
| --- | --- | --- | --- | --- | --- |
| Alabama | 132.31 | 121.44 | Montana | 34.55 | 30.38 |
| Alaska | 38.57 | 37.90 | Nebraska | 49.43 | 49.87 |
| Arizona | 93.93 | 89.68 | Nevada | 37.01 | 33.89 |
| Arkansas | 65.59 | 65.05 | New Hampshire | 16.42 | 14.19 |
| California | 360.31 | 344.94 | New Jersey | 110.68 | 99.08 |
| Colorado | 95.06 | 89.43 | New Mexico | 54.22 | 54.22 |
| Connecticut | 35.11 | 32.25 | New York | 170.07 | 154.29 |
| Delaware | 11.09 | 12.84 | North Carolina | 138.74 | 114.81 |
| District of Columbia | 3.22 | 2.61 | North Dakota | 52.13 | 55.89 |
| Florida | 239.16 | 218.28 | Ohio | 246.39 | 213.09 |
| Georgia | 169.70 | 133.21 | Oklahoma | 105.61 | 104.14 |
| Hawaii | 18.86 | 18.73 | Oregon | 39.81 | 36.03 |
| Idaho | 15.76 | 15.20 | Pennsylvania | 253.27 | 233.37 |
| Illinois | 228.36 | 212.26 | Rhode Island | 10.76 | 10.27 |
| Indiana | 216.02 | 191.79 | South Carolina | 82.75 | 71.67 |
| Iowa | 87.23 | 77.53 | South Dakota | 14.96 | 14.68 |
| Kansas | 75.22 | 67.96 | Tennessee | 107.50 | 96.97 |
| Kentucky | 150.30 | 136.83 | Texas | 662.35 | 656.39 |
| Louisiana | 227.80 | 211.71 | Utah | 63.57 | 60.97 |
| Maine | 17.83 | 15.51 | Vermont | 5.76 | 5.32 |
| Maryland | 68.86 | 58.60 | Virginia | 107.00 | 95.06 |
| Massachusetts | 70.41 | 59.02 | Washington | 74.20 | 69.06 |
| Michigan | 163.15 | 150.28 | West Virginia | 99.03 | 90.80 |
| Minnesota | 91.86 | 85.24 | Wisconsin | 97.55 | 88.39 |
| Mississippi | 65.24 | 60.43 | Wyoming | 65.04 | 66.10 |
| Missouri | 133.71 | 124.94 |  |  |  |
